# ‘You have a child who will call you “mama” ’: understanding adolescent pregnancy in South Sudan

**DOI:** 10.1080/16549716.2018.1553282

**Published:** 2019-01-08

**Authors:** Sumit Kane, Esther Miedema, Marjolein Dieleman, Jacqueline Broerse

**Affiliations:** a KIT Health, KIT Royal Tropical Institute, Amsterdam, The Netherlands; b Nossal Institute for Global Health, Melbourne School of Population and Global Health, University of Melbourne, Melbourne, Australia; c Governance and Inclusive Development Programme, University of Amsterdam, Amsterdam, The Netherlands; d Faculty of Earth and Life Sciences, Athena Institute for Research on Innovation and Communication in Health and Life Sciences, VU University Amsterdam, Amsterdam, The Netherlands

**Keywords:** Adolescent pregnancy, teenage pregnancy, reproductive health, South Sudan, reproductive agency

## Abstract

**Background**: Pregnancy amongst adolescent girls is common in many parts of the world. The dominant discourse in public health unquestioningly paints this as a problem; it does not pay sufficient attention to girls’ views.

**Objectives**: This paper presents a critical account of adolescent South Sudanese girls’ reasons for and explanations of childbearing. It discusses their experiences and views on childbearing and attempts to explain their reproductive choices and actions, in context.

**Methods**: The study draws upon 24 interviews with adolescent boys, girls and parents from Wau, South Sudan. Data was analysed using the framework analysis approach.

**Results**: Three interacting themes within which adolescent girls framed their views and decisions about childbearing are identified. The local society places high value on motherhood – adolescent girls’ desires to become mothers is a reproduction of this social norm. Girls linked having a child to the possibility of making one’s ‘own home’; in the difficult and uncertain context they lived in, for many girls, having a child (and making a home) appeared as one of the few means to be happy. In making the decision to bear a child, the girls navigated multiple dilemmas and trade-offs between an unpromising present and an uncertain future. Bearing a child and making one’s ‘own home’ was seen as a way to exit into the world of adults, and as a strategy towards achieving security and stability.

**Conclusions**: Instead of simplistically problematizing adolescent pregnancy in South Sudan, it is important to take into account the experiences and standpoints of adolescent girls, and to recognize that in choosing to become mothers, they are in many ways exercising agency despite being severely constrained by complex, insecure and unfair social circumstances. We argue that such an approach will allow the development of more appropriate, realistic and inclusive health and social policies and programs.

## Background

South Sudan has experienced war and civil unrest for many decades now. As a result, it features low on all global health and development performance indices. In terms of sexual and reproductive health (SRH), South Sudan’s challenges are particularly telling. Almost half of its population is below 20 years of age, and as UNICEF’s *State of the World’s Children* report of 2016 highlights, a disproportionate part of this burden is borne by adolescents, particularly by girls [1]. According to the national family planning policy of South Sudan, ‘by the age of 19, one out of three girls is already married or in union; and the same proportion has already started childbearing’ [2]. There is robust evidence that in contexts like that of South Sudan, pregnancy and childbirth carry higher risks during adolescence, with risk of mortality being almost twice as high when compared to women in their twenties; adolescent mothers also have a higher risk of non-fatal pregnancy- and childbirth-related complications; similarly, the risks of low birth weight, stillbirth and neonatal death are also greater among adolescent mothers [3–5].

In view of these concerns, and given the evidence, the family planning policy of South Sudan explicitly seeks to ‘promote an enabling legal and social-cultural environment that ensures individuals, especially women and girls are able to claim and exercise their rights’ [6]. International organizations, key actors in the public health arena in South Sudan, also echo these concerns; they have regularly called for, and resourced, public policy and program responses to tackle early marriage, early childbearing and the associated health problems in South Sudan [1,7,8]. Many studies and reviews have highlighted that social and economic factors are key drivers of adolescent pregnancy [9–13]. In their comprehensive review, Mc Queston et al. [10] argue that ‘to the extent that adolescent fertility is symptomatic of deeper factors, policymakers should focus on the distal rather than immediate causes of early childbearing’ (p.45). One of the key insights they arrive at is that attributing pregnancies amongst adolescents to lack of knowledge about SRH or a lack of economic resources, as many are often wont to, is simplistic. They argue that often girls’ desires to have children are socially constructed and rooted in social pressures to become pregnant; and that they are often shaped by a social context which either offers no incentives to delay fertility, or, conversely, offers incentives to bear children [14,15].

Echoing Mc Queston et al.’s [10] conclusions, through this paper we argue that public policy responses, including the reproductive health policy responses, would be more effective if the reasons for early childbearing amongst adolescent girls in South Sudan were better understood, and better taken into account. Crucially, as Heavey et al. argue [14], current sexuality education interventions tend to be grounded in the notion that adolescents are becoming pregnant by mistake and that more information about, and access to, contraceptives will lead to a reduction in pregnancy rates among adolescents (see also Davies et al., 2008 [15]). In this regard, feminist scholarship is critical to the current debate, particularly that which has highlighted the normative underpinnings of SRHR initiatives. As authors such as Mann (2013) [16] contend, SRH interventions geared at economically disadvantaged young women and men are often underpinned by discourses of deviance and normalization and need to be understood as sites for regulating young women’s sexuality. Building on these authors, we argue that in contexts where adolescent girls desire pregnancy, current approaches to preventing adolescent pregnancy are not only likely to continue to fail, but also potentially do harm by implicitly framing adolescent mothers as deviant. More texturized understanding of adolescent girls’ motivations with regard to childbearing is crucial to developing not only more ‘effective’ but also more inclusive programs.

However, bar research by the authors mentioned above, little is known about adolescent girls’ desires to bear children, particularly in sub-Saharan Africa. In this paper, we seek to provide a hitherto unreported account of adolescent South Sudanese girls’ reasons for and explanations of childbearing. We analyze adolescent South Sudanese girls’ views on pregnancy and the value they attach to childbearing in an effort to understand the critical drivers of pregnancy among adolescent girls. The perspectives of parents and adolescent boys are used to nuance this understanding. In doing so, we reflect upon the implications of this insight into sexual and reproductive health policy and practice in South Sudan. This paper presents findings from a qualitative study conducted within the context of the South Sudan Health Action and Research Project (SHARP) project in Wau Town, Western Bahr el Ghazal State (WBeG) of South Sudan. The project was implemented between 2012 and 2016 and was geared towards supporting the State Ministries of Health of three states (including WBeG) to improve the quality and responsiveness of the sexual and reproductive health services. Findings from research done amongst adults have been reported earlier [17–20]. Insights from this research, particularly about how social norms and gender relations shape women’s sexual and reproductive health in South Sudan, have also extensively informed this inquiry; it particularly helped us to develop the interview questions and to locate the analysis in the broader social context of the study community.

## Methods

Given the focus on gaining insight into adolescent girls’ reasons for and explanations of childbearing, an exploratory qualitative study was conducted. Data was collected through in-depth interviews conducted with purposefully selected participants, as detailed in Table 1. Interview topic guides were developed based on the insights gained from earlier work conducted amongst adults [17–19], and included questions exploring norms and beliefs about sex, sexuality and childbearing, and on what shaped these amongst adolescents. Those between 15 and 20 years of age were considered as adolescents for this study; this was consistent with the local adolescent reproductive health policy (it was being developed at the time of the study). In this paper, however, the terms ‘girl’ and ‘young woman’ are used interchangeably; this mirrors how the study participants referred to themselves. The topic guides were defined further during the initial stakeholder consultations, pre-tested in the study site, and were also adapted iteratively as the study progressed. The topic guides were prepared in English and translated into Wau Arabic. The interviews were conducted in Wau Arabic, a language spoken by all around Wau town.

**Table 1.** Study participants.

Interview participant profile	Numbers
Adolescents	
Female in school – with child	2
Female in school – no child	5
Female not in school – with child	4
Male in school – no child	4
Male in school – with child	2
Male not in school – with child	4
Parents	
Parent	3
Total	24

### Sampling, recruitment of study participants and data collection

Based on the recommendations of the scientific and ethical review group’s observations on reproductive health research involving adolescents [21], boys and girls between 16 and 20 years of age were included as participants. Adolescents were purposefully selected to capture diverse experiences and perspectives. Based on earlier research and on a review of the literature it was deemed that having children or not, and whether one had to forego schooling as a result of having a child or not, were important criteria – having (or not) these experience would allow the adolescent to look at the phenomenon from a unique perspective. We thus selected adolescents who were in school and also those not in school; and those with children, and those without children, for inclusion in the study. Adults, who were parents of adolescents with children, were also interviewed to understand their perspective. Participants were recruited from Wau town with the help of a local youth outreach worker and through a snowball sampling approach. The school-going adolescents were studying in grades 6 to 9. Interviews were conducted on the premises of a residential school for nurses as its location was well known to all, and it offered privacy. Potential participants were approached in the community, the study was explained to them, and they were asked to come to the venue if they were interested in being part of the study. Of the 25 adolescents approached, 20 were ultimately included in the study – 3 did not show up at the appointed time; 2 arrived late and chose to leave as they could not wait.

Data were collected between April 2014 and November 2015. Interviews were conducted by research team members who were fluent in Wau Arabic and had experience with conducting interview-based qualitative research; interviews typically lasted for 60–75 minutes. Data were collected till analytical saturation was reached, and no new insights emerged; this was possible to assess, as at the end of each day of data collection, the research team debriefed and discussed the emerging findings. In total 24 interviews were conducted.

### Data analysis

Interviews were digitally recorded, translated from Wau Arabic into English, and transcribed verbatim (with assistance from two local research assistants). A framework analysis of the data was conducted as per Ritchie and Spencer [22]. The authors (SK, EM) read and discussed some of the interview transcripts to inductively identify key issues and themes in the data; together with SK’s field notes, an a priori thematic framework was developed. This thematic framework was used to analyze all the transcripts – this entailed indexing, charting and mapping of the transcripts to the themes included in the a priori thematic framework. This was done using NVivo 11 software. If new issues came to our attention, they were added to the thematic framework; similarly, the a priori themes were also modified/nuanced as appropriate. The analysis and interpretation of the text coded to the themes was done through a process of discussion and argumentation (SK, EM); it also entailed linking the analysis to existing social theory to arrive at explanations. This process continued iteratively through to the writing up of the analysis and the drafting of this manuscript.

To ensure credibility [23] an explicit and ongoing attention was given, both during data collection and during analysis, to disconfirming, paradoxical and counterintuitive findings; this enabled the refinement and contextualization of findings. This was done through a process of argumentation and discussion: initially during the daily post-data collection debriefings, later during a data analysis workshop, and on an ongoing basis via email during the process of writing the analysis. To ensure dependability [22], on various occasions, the findings, interpretations and conclusions were cross-checked with three people who were knowledgeable about the study community.

## Results

We identified three interacting themes within which adolescent girls framed their views and experiences about childbearing. The first theme relates to the various ways in which girls, and the society at large, value motherhood. Linked to the first theme, the second theme relates to how having a child is a fulfilling experience for adolescent girls, particularly in combination with making one’s ‘own home’. For many girls, having a child (and making a home) appeared one of the few means to be happy, in the difficult and uncertain context they lived in. This notion of ‘home-making’ emerged as a major and multifaceted theme that variously related to the first two themes; the first two themes also serve as the backdrop for this third theme. Various facets of what the notion of ‘home-making’ means and connotes to girls are examined. In doing so, the third theme locates girls’ decisions to bear a child and to make a home as processes of navigating dilemmas where girls make very personal trade-offs between an unpromising present and an uncertain future.

## The entry into adulthood: motherhood (and fatherhood)

Girls’ accounts of the value of motherhood, including their own motherhood, and how having a child was seen amongst their peers and by their parents, reveal that having a child seems to somehow define one’s worth and establish one’s claim to adulthood. Amongst the study participants, the girls who had children of their own, whether they were in school or not, seemed to take much pride in being mothers. This pride was akin to a certificate of honor, marking entry into adulthood, as mentioned in the quote below:

They [the girls who have children] tell us that we do not have any value because you do not have children. It is better to have a child. This way you will be considered to have two certificates, one for the school and another for motherhood. They also tell us that having your own home is the best. [Girl in school – no child – 2]

The following quote by a young mother illustrates how motherhood is also seen as signaling entry to womanhood and into the world of adults and how it somehow entitles one to have a say in the community. This notion of having a child and being responsible for the child was a common narrative among young mothers. Young women’s accounts suggest that having a child was not seen as being burdened with responsibilities, but rather about being seen as someone who was capable of shouldering responsibility as an adult, and as someone able to guide her children:

It [becoming a woman] means to become a mother, and she guides her children and people in the community. [Girl in school – with child – 1]

This attachment of high value (amongst girls) to childbearing, was consistent, as reported earlier in a study amongst adults, with the local social norms [17,18]. One can argue that in saying the above, the girls was merely conforming to particular social norms, and perhaps mimicking what they saw the adults around them value. The adults’/parents’ views were mixed; the following quotes from the mother of a girl (a girl who got pregnant while still in school) show that, even as she acknowledged that a girl’s pregnancy is appreciated in the community, she wanted her daughters to focus on their education:

It [one’s child becoming pregnant] is seen as a good thing especially given her young age. … I expect my daughters to get a good education and leave all the nonsense alone. [Mother of girl in school – with child]

While to an outsider these views might appear to be contradictory, to the parents themselves it was only problematic if the man who had made the girl pregnant did not take responsibility and did not provide for the girl and the baby. In the case of the parent who is quoted above, at the time of the interview her daughter was pregnant again, by the same man. To us it appeared that as long as the man supported the girl socially and financially (even if he did not marry her or take her to his house, as is customary), the girl’s mother (and the girl’s father alike) had no problems with looking after her daughter and her grandchild. The girl’s mother also had no problems with keeping the two (her daughter and the grandchild) in her own house – in fact she was keen on helping her daughter to finish her schooling. As the following quote shows, this interpretation echoes what the young men in this study said about what was expected of them by society:

By God, if you beat her or impregnate and leave her. By that, her family becomes angry, or she becomes angry too. Then they say that the boy has impregnated her and left her, you see how the boys … behave […] Yes, if you impregnate her and take her responsibility … maybe people can be happy with you. [Young father not in school – 1]

All the young men in the study equated fatherhood, with being and becoming a man, and being considered a ‘good man’, with being a ‘responsible man’. Also, all participants in this study, including all the young men, felt that a responsible man, however young he might be, was someone who would step up and accept responsibility if he had impregnated someone – and would provide for the mother and the child. All the young men reported that if one did not take care of the mother and the child, it was a matter of shame. This was so much so that many young men had fled the town because they could not (or did not want to) fulfill their paternity responsibilities.

## The value of motherhood

### Motherhood: a meaningful and fulfilling experience

For girls, whether they were in school or not, having a child of one’s own was important. As the following quote from a young mother illustrates, it was seen as a source of fulfillment and it added a sense of meaningfulness to an uncertain present and unpredictable future:

Participant: It means that you have a child who will call you ‘mama’.

Interviewer: Can you explain?

Participant: It can be next to you … will listen to you, you play with it, and will be in your life always and will call you my mother and so on. [Girl not in school – with child – 3]

While on one hand young women reported experiencing pride, and a sense of fulfillment and purpose, on having children, many young mothers also consistently reported being disappointed with their lot. This tension between, on one hand, the desire and need to fulfill one’s own and to some extent social expectations related to fertility, and the belief that having children was essential for attaining adult status, and, on the other hand, the price one had to pay for early childbearing, specifically in terms of dropping out of school, was a dilemma all girls seemed to grapple with. This tension was clearer in young mothers’ descriptions of their lives, particularly of those who had to drop out of school as a result of becoming pregnant. The following excerpt from a young mother who had to drop out of school reflects her disappointment, worry and despair, including, but not limited to, about having to forego school:

Participant: There are those who feel happy and others don’t.

Interviewer: Why will one not feel happy?

Participant: How am I going to think about the future of my child and my studies, and my child’s schooling, when he grows up, he wants to eat, drink, clothes […] I feel that who will stand by my side when my child is big … then nobody would stand beside me and … [Girl not in school – with child – 2]

While the young men in the study did not have much to say about how they thought the girls related to and experienced being pregnant and having children, their views on being a father were mixed. As the excerpt from a young father shows, for some it was clearly important, but for some, not so:

Some love to be called dad but other don’t want that. Some say if you don’t have a child when you die, god is going to ask you that … he sent you to the earth and what have you done for him. [Young father not in school – 3]

### Motherhood: carrying one’s name forward

A further point that underscored the value of having a child related to the cultural notion of having someone to carry one’s name after one dies. The girls in the study consistently talked about how having a child ensured that their name would not be forgotten; a common reasoning across the girls interviewed was that it was better to have a child as soon as possible, lest one was to die prematurely – a very real risk in the context of South Sudan:

Yes, they see it as becoming famous. Some also say that there is premature death that can come unexpectedly, so it is better to have a child so that if I die my name will not be forgotten. When they see my child, they will say this is so and so’s child. [Girl in school – no child – 1]

This finding perhaps represents a shift of sorts, in the local social and gender norms around who has a claim to the child and whose name the child carries into the future. In the local society, the family is a consanguinal unit built around a core of brothers and sisters (blood relations); the wife is not seen as part of the family, and her role is to bear children for the man’s family, ostensibly to carry the man’s and his family’s name forward [17]. While in our earlier work with adults [17,18], women and men were very clear about who had the claim on the child (the man and his family), and whose name the child would carry into the future (the man’s), the girls in this study saw the child as very much their own and someone who would carry their name forward.

## Home-making

### Home-making: a means to stability, self-worth and happiness

Those who leave school think that the most important thing is to have their own home […] as a wife and mother. [Girl in school – no child – 1]

Instead of staying at school all day and not having money for breakfast it would be better to become pregnant and stay home […] this is what most girls are saying. [Girl in school – no child – 5]

As the above quotes allude to, the notion of having a ‘home’ was integral to young women’s narratives as to what it meant to become a woman and establishing a family and life of their own. The excerpts above, from two school girls (with no children), similarly highlight the centrality of ‘home-making’ and all it entailed – having a child, a husband and thereby social standing as a wife and mother – in young women’s lives. As another young school-going woman explains when asked as to how young women in her surroundings perceived the role of a woman,

Girls think that becoming a woman means that you will be happy in your home and you will not need anything. You will have enough money and you will have the say in the home. [Girl in school – with child – 2]

The quote highlights the view amongst girls that creating one’s own home will offer them not only financial stability but also a measure of independence, voice and, ultimately, happiness that they may not have in their current home, either parental or otherwise. A young father similarly engages with the idea of stability, as well as alluding to the pressures of ‘people’ on young women who had not yet established their own homes:

For an unmarried girl, she has no husband any way. […] There are people who insult her that, [saying:] ‘Aha!’ […] She is useless […] if not she should have been made stable at home by someone and have children. [Young father in school – 1]

### Home-making: balancing between peer and social pressure

The above quote (from the young father) signals that a young woman who had no children or had not yet found someone to ‘make her stable,’ was regarded as not having any ‘use’ or worth. However, it was unclear as to whether this ‘uselessness’ was configured in relation to the community or a particular group; for example, certain peers (young women who had children or young women of a certain age). Participants often alluded to the community not ‘wanting girls to hurry to the extent of getting pregnant and deliver earlier’, but instead, resources permitting, to ‘get educated, obey their parents, and not get into problems’, such as pregnancy. The following excerpt from an interview with the same young school-going woman as cited at the beginning of this section (on home-making) is illustrative of the importance young women appeared to attach to creating a home, as well as speaking further to the notion of ‘pressures’:

Interviewer: [You indicated that] young women at your age [who] don’t want to continue with their school, the most important thing for them is their home. [Where] does this thought come from?

Participant: This thought comes when they are not happy together at home[…] This sometimes brings about this thought or when she is given much pressure. […] This is when she is having lots of problems at home […]. The problems can be like abuse, for example, or much pressure. [Girl in school – no child – 1]

When asked about the pressures a family might exert, the young woman explained that it was pressure to

pay attention to herself […] so that she thinks about her future not anything else, […] like the street, random movements, or bad life behavior, […] life … early marriage. [Girl in school – no child – 1]

The excerpts above and those in the previous sub-section together suggest several interrelated issues. They show young women’s struggles in navigating competing social pressures and claims about the appropriateness of their reproductive choices and life decisions. On one hand, there is pressure from relatives and family members warning them to ‘stay far away from boys’ following menarche, and from community members expecting them to complete their education first. On the other hand, there is the peer pressure to obtain the ‘two certificates’ mentioned earlier, and to make one’s own home. These narratives of competing social pressures resonated with the accounts provided by other young women in our study.

### Home-making: reinterpreting the notion of marriage

A second salient issue with regard to some of the extracts given above concerns the question of marriage. According to the young women and men involved in the study, caregivers and the broader community encouraged young people to delay pregnancy (and by implication sexual relations) until *after* marriage. ‘Marriage’ then seems to be conceived in the conventional sense; that is, as a ceremony marking the formal process of social approval of conjugal relations between a man and a woman. However, as some of the extracts given above illustrate, in practice, the point at which young people deemed themselves to be ‘married’ and became a husband or wife was more loosely defined by young people and to some extent also by society at large; it related to when a young woman/couple became pregnant. The following interaction with a young father shows how the notion of a wife probably had more to do with a girl being the mother of one’s child, and not necessarily about someone one is ‘married’ to in the traditional and conventional way – when the interviewer asked about whether boys wanted to be fathers, the young man’s response is about wanting to have a wife:

Interviewer: Are there boys from your group who want to be a father?

Participant: Yes, it is there, one wants to have a wife … so as to have a child, so that he grows up with him together. [Young father not in school – 2]

As another young school-going woman relates,

Most girls after menarche see themselves as she can do anything, she feels like she is a woman and can handle a home. […] When a girl becomes pregnant she sees herself as a housewife […]. She will feel that she has to become responsible, she has become a house wife who needs to raise her child. [Girl in school – with child – 2]

When asked whether all girls who got pregnant became ‘house wives’, the young woman clarified that ‘no, […] some end up staying with their parents’. The notion of ‘house wife’ appears to be used synonymously with ‘wife’, with the quotes suggesting that a young woman (and also likely applicable to young men) was only considered ‘married’ when living with the father (or mother) of the child, be it in his family home or ideally – as participants’ accounts suggest – in their own home. Young people, and young women in particular, thus seemed to hold opposing views regarding the desirable route to adulthood when compared with caregivers and the broader community. Whereas the latter set of actors sought to encourage girls to complete their education, and only then marry and have children, many young women regarded pregnancy as a pathway to establishing their own home with a husband and child. It was this set-up that was defined as marriage, and which was seen as offering a means to create (greater) stability and potentially happiness in their lives. As the quote above also indicates, in the local society, menarche thus signals a defining moment of agentic possibility for young women; and getting pregnant, and making a home, are expressions of this agency – the former being a cause for what appears to be considerable concern for parents and health services, but perhaps less so for peers and also the society at large.

## Discussion and conclusions

The data presented here suggest that for adolescent girls involved in this study, having a child has multiple meanings and represents an attainment – a ‘certificate’ of much importance. Within adolescent peer circles, and to some extent in the society, in the study area, childbearing signals worthiness – and while our data do not allow a complete understanding of what all this ‘worthiness’ entails, it does appear to be related to demonstration of one’s childbearing potential. Childbearing also seems to provide meaning and satisfaction in achieving something that is valued highly in society, in a context where prospects of achieving something socially valuable through other means are very few. To adolescent girls, and, while it does not emerge explicitly from our data, to adolescent boys too, having a child is also seen as a ‘ticket’ into the world of adults – having borne a child, irrespective of whether one is married or not, almost appears to be a proxy for being a respectable and responsible adult. Similar findings have been reported by Gyesaw and Ankomah [24] from Ghana, and by Goicolea [25] in her research amongst adolescent girls in poor and violent communities in Ecuador. Goicolea shows how for some girls, motherhood served as a ‘safety-belt’, helping protect them from physical and reputational violence. In our study, for adolescent girls, motherhood opened the prospect of exit from households where they were unwelcome and/or were in penury; by becoming mothers they could exit to the security and dignity of their own home that they would make together with the father of the child. It follows that given this complex determination, a nuanced view of adolescent pregnancy in South Sudan at public policy and program levels is warranted.

Having children, many children, is socially desirable in South Sudan. As reported elsewhere, by us [17] and by Elmusharaf et al. [26], for a variety of reasons, great value is placed on a woman’s ability to bear children. While adolescent girls’ decisions to bear children is consistent with, and in some ways a reproduction of, this social norm, our findings show that these decisions are also shaped by the harsh economic and social realities which constrain the futures the girls may be able to imagine for themselves. A bleak present and a paucity of viable prospects for the foreseeable future can perhaps also explain why girls choose to get pregnant, somehow betting on and hoping for some financial security from the father, in spite of the expectations of their parents and other members of society to the contrary. This exercise of agency by adolescent girls was variously constrained and entailed many personal trade-offs. Between having food and social security now, and hunger and social insecurity now and in the foreseeable future. Between being unwelcome in their current household, and the possibility of leading a dignified life as a wife/mother (traditional forms of formalization of marriage being not necessary, merely being taken in by a male provider being enough). These findings are consistent with a recent survey-based study on adolescent pregnancy in Juba, the capital of South Sudan [27].

In the post-war context of South Sudan, the educational system has broken down, while economic opportunities generally, and prospects for adolescents and young adults in particular, are few. In addition, the civil conflict in the last decade has led to widespread displacement, and this has meant that many adolescents have been raised in households with little resources and multiple claimants on these limited resources. While none of the study participants explicitly mentioned that they were unhappy, the desire to leave their current homes, and to forge their own homes, was a consistent theme. Given this context, Hagan and Wheaton’s [28] argument that ‘researchers give more attention to linkages between particular behaviors within the larger theoretical context of the life course and the social roles of adolescence and adulthood’ (p.955), as a frame can help provide a more meaningful explanation and understanding of adolescent girls’ desire to have children. It helps one recognize that adolescents’ desire to have children themselves, and their enactment of these desires, might be understood as attempts to escape from the category of the child, and to seek early entry into adulthood. This ‘exit’ perspective can also be understood in relation to the body of evidence [29] demonstrating that the presence of ‘aspirations’, and the prospects of opportunities to fulfill these aspirations, are important determinants of adolescents’ reproductive decisions. Such an understanding is consistent with research amongst adolescents in poor and marginalized communities in other parts of the world. Dunn [30] found that for many adolescent women in inner-city Washington, DC, becoming a mother was the most viable and socially rewarding situation to be in. Bennett [31] in her work amongst adolescent mothers in poor communities in Lombok, Indonesia, extends this understanding to argue that in contexts of entrenched poverty and poor economic prospects, for girls and their families, early marriage and childbearing are a way to assure economic safety. Our analytical inference thus leads us to the argument that it is important to pay attention to whether ‘adolescent pregnancy causes poorer life prospects or if poor life prospects motivate early pregnancy’ [9]. Finally, the exit perspective implies that if social and developmental interventions work to create opportunities and lower the barriers for adolescent girls to imagine and strive towards achievable futures, they will make different choices, irrespective of whether they currently have children or not. This analysis is in line with the evidence that shows that interventions that focus on inclusive educational, economic and social development can be effective in alleviating the structural disadvantages that beget adolescent childbearing [9,27,32].

At a different level, how pregnancy, childbearing and parenthood are viewed by individuals, and what value is attached to them, at different stages in one’s life as a social being, is a function of the social and gender norms of a particular society [33,34]. Therefore, it is not appropriate to examine and to simplistically judge the childbearing- and motherhood-related standpoints of adolescent girls of Wau as ‘problematic’. Doing so negates the centrality of childbearing and motherhood in the local social and relational context. Doing so also entails unquestioningly assigning victim status to the young women; who, as our findings show, clearly cherish and actively seek motherhood. Our findings echo the conclusions of Holgate & Evans [35]; in their authoritative book examining teenage pregnancy and parenthood, they contend that ‘the conceptualisation that there is a “right” time and a “right” framework in which to mother reflects an oversimplification of the complexities of motherhood. Perceptions of what constitutes the “right” time and framework are historically and culturally specific.’ Like us, they urge policy makers to recognize the significance of the contexts of class and socio-economic status in which ‘all women mother’. Going further, examining experiences and standpoints of adolescent girls of Wau through an African feminist perspective [33] allows one to recognize that, despite being severely constrained by complex insecure social circumstances, girls are, in important ways, exercising agency. These findings are consistent with research on early marriage and early childbearing amongst Syrian refugees, who in many ways are in a similar complex and insecure social circumstance [36]. Extending Vincent and Alemu’s [27] point, we argue that public health policy makers should make conscious efforts to better understand the experiences and standpoints of adolescent girls, and recognize that in choosing to become mothers, many are exercising agency in complex and unjust social circumstances. Understanding adolescent girls’ motivations will enable policy makers to develop health and social policies and programs which are realistic, and which genuinely contribute to the physical, mental and social well-being of adolescents.

Specifically, for South Sudan, social and health development interventions should focus on simultaneously reconfiguring the various aspects of this complex sociality, and on enabling adolescent girls to navigate it, in ways which enable them to imagine the futures they desire, and to exercise agency towards achieving these futures. While it is beyond the scope of this paper to delve into details, drawing on recent reviews [9,32], two social policy avenues deserve attention. One way forward would be to work with the school system to support young mothers and to ensure that they can continue to study and can make informed choices about further childbearing. Such an approach may also offer opportunities to engage constructively with peer norm processes among girls (adolescents and younger girls). Another important way forward, as detailed in our earlier work [20], would be to engage adolescent boys and men around notions of reproductive responsibility in ways such that they can appreciate the broader societal benefits, and their own ‘emancipatory interests’ [37,38].

## Limitations

The study has limitations. Our study participants were people from a poor socio-economic background; while one could argue that this would represent the vast majority of the people in the study area, it is very likely that the findings do not capture the lived realities of adolescents from better-off and better-educated, stable families. Similarly, all the girls in the study lived either with a parent or with the family of their child’s father. Again, while this is the norm, given the context of South Sudan, there are many adolescent-headed households – our findings may not apply to the lived realities of such adolescents. The analysis presented in this paper also has limitations – these are primarily a result of the choices we have made in writing the paper. Our data lends itself to being interrogated from other important perspectives too; for instance, analyzing the data to expose and explicate how gender power relations shape adolescents’ actions would yield valuable, policy-relevant insight.

## Paper context

Globally, in public health policy and practice, pregnancy amongst adolescent girls is commonly presented as a problem related to girls being young, uninformed and helpless. This study presents a critical and nuanced account of adolescent girls’ reasons for and explanations of childbearing. By spotlighting girls’ explanations of their reproductive choices and actions, the study shows how many girls choose to become mothers, exercising agency, in what are often very complex and unjust social circumstances.
